# A Brief Assessment of Intelligence Decline in Schizophrenia As Represented by the Difference between Current and Premorbid Intellectual Quotient

**DOI:** 10.3389/fpsyt.2017.00293

**Published:** 2017-12-22

**Authors:** Kazutaka Ohi, Chika Sumiyoshi, Haruo Fujino, Yuka Yasuda, Hidenaga Yamamori, Michiko Fujimoto, Tomiki Sumiyoshi, Ryota Hashimoto

**Affiliations:** ^1^Department of Neuropsychiatry, Kanazawa Medical University, Uchinada, Japan; ^2^Faculty of Human Development and Culture, Fukushima University, Fukushima, Japan; ^3^Graduate School of Education, Oita University, Oita, Japan; ^4^Department of Psychiatry, Osaka University Graduate School of Medicine, Suita, Japan; ^5^Department of Clinical Epidemiology, Translational Medical Center, National Center of Neurology and Psychiatry, Kodaira, Japan; ^6^Molecular Research Center for Children’s Mental Development, United Graduate School of Child Development, Osaka University, Suita, Japan

**Keywords:** schizophrenia, intelligence decline, premorbid intellectual quotient, current intellectual quotient, Wechsler Adult Intelligence Scale

## Abstract

Patients with schizophrenia elicit several clinical features, such as psychotic symptoms, cognitive impairment, and subtle decline of intelligence. The latter two features become evident around the onset of the illness, although they may exist even before the disease onset in a substantial proportion of cases. Here, we review the literature concerning intelligence decline (ID) during the progression of schizophrenia. ID can be estimated by comparing premorbid and current intellectual quotient (IQ) by means of the Adult Reading Test and Wechsler Adult Intelligence Scale (WAIS), respectively. For the purpose of brief assessment, we have recently developed the WAIS-Short Form, which consists of Similarities and Symbol Search and well reflects functional outcomes. According to the degree of ID, patients were classified into three distinct subgroups; deteriorated, preserved, and compromised groups. Patients who show deteriorated IQ (deteriorated group) elicit ID from a premorbid level (≥10-point difference between current and premorbid IQ), while patients who show preserved or compromised IQ do not show such decline (<10-point difference). Furthermore, the latter patients were divided into patients with preserved and compromised IQ based on an estimated premorbid IQ score >90 or below 90, respectively. We have recently shown the distribution of ID in a large cohort of schizophrenia patients. Consistent with previous studies, approximately 30% of schizophrenia patients had a decline of less than 10 points, i.e., normal intellectual performance. In contrast, approximately 70% of patients showed deterioration of IQ. These results indicate that there is a subgroup of schizophrenia patients who have mild or minimal intellectual deficits, following the onset of the disorder. Therefore, a careful assessment of ID is important in identifying appropriate interventions, including medications, cognitive remediation, and social/community services.

## Intelligence Decline (ID) in Schizophrenia

Schizophrenia is a common and complex psychiatric disorder with clinical and genetic heterogeneity (1). The lifetime risk of the disorder is approximately 0.5–1% (2). The disorder is characterized by a wide spectrum of symptoms, such as delusions, hallucinations, blunted affect and withdrawal, cognitive impairments, as well as subtle decline in intelligence. Cognitive impairments in numerous and diverse domains, including attention, working, verbal and visual memories, processing speed, social cognition, and general intelligence (i.e., a 1- to 2-SD decline in performance on neuropsychological tests compared with healthy individuals), are a core feature of the disorder and a reasonable target for treatment (3–9). These deficits contribute to social or occupational dysfunction and poor life outcomes (10–12). Cognitive impairments and psychotic symptoms are relatively independent dimensions of the disorder (13). Cognitive impairments are exhibited around or after the onset of schizophrenia, while, in a substantial proportion of cases, the impairments exist even before the disease onset (14–16). On the other hand, intelligence decline (ID) represents intra-individual differences in intellectual quotinent (IQ) at different time points, such as before and after the onset of morbidity (13, 17, 18). In this article, we review the literature concerning ID during the progression of schizophrenia.

## A Brief Assessment of ID in Schizophrenia

**Figure 1:**
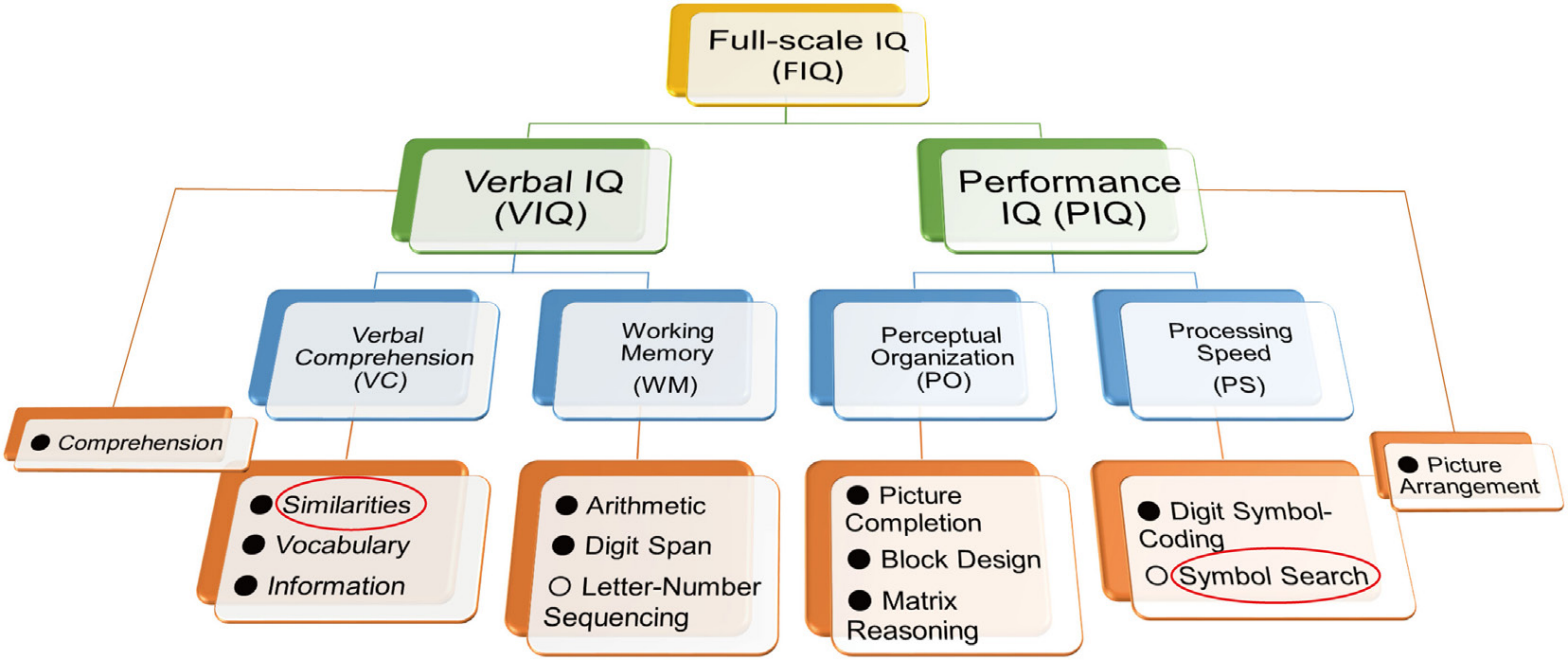
Structure of the Wechsler Adult Intelligence Scale (WAIS)-III. To shorten administration time and assess functional outcome in patients with schizophrenia, we previously developed the WAIS-III Short Form using the Similarities and Symbol Search components (red circles) (23). The administration time was shortened to approximately 10 min. Letter-Number Sequencing and Symbol Search (○) are not used for calculation of verbal intellectual quotient (VIQ) and performance intellectual quotient (PIQ), respectively.

Intelligence decline is defined as a decrease in current intellectual quotient (IQ) from a premorbid level in patients with schizophrenia (13, 17, 18). ID can be estimated by comparing standard assessments of estimated premorbid and current IQ using the Adult Reading Test and the Wechsler Adult Intelligence Scale (WAIS), respectively. The WAIS has been widely used to measure current intellectual performance in patients with psychiatric disorders as well as healthy subjects. The battery has been updated several times [WAIS (19); WAIS-R (20); WAIS-III (21); and WAIS-IV (22)]. To represent the intellectual construct in a healthy subjects, the four factors, Verbal Comprehension (VC), Working Memory (WM), Perceptual Organization (PO), and Processing Speed (PS), were established in the WAIS-III (Figure 1). VC and WM are components of verbal IQ (VIQ), while PO and PS are components of performance IQ (PIQ). In the updated WAIS-IV, the dual IQ (VIQ and PIQ) scoring system was eliminated, and the concept of index-based assessment of intelligence has been further enhanced. Furthermore, two subtests (Object Assembly and Picture Arrangement and) in the WAIS-III were replaced by newer subtests (Figure Weights and Visual Puzzles) in the WAIS-IV to enhance psychometric validity and user friendliness (23).

It takes 60–95 min to administrate the WAIS in healthy subjects. As it has been concerned about the lengthy administration time for the WAIS, it has been made efforts to develop the WAIS-Short Form (WAIS-SF) (23–26). The typical approach to developing the WAIS-SF was to select subtests to optimize the prediction of full-scale IQ (FIQ) and/or retain the representativeness of the IQ structure (26). However, it is presumed that the four-factor structure for healthy subjects does not need to be preserved in patients with schizophrenia (23, 26); e.g., if VC and WM were collapsed into a single factor in patients with schizophrenia, selecting a subtest from each of these domains would be redundant (26). Another problem for previous WAIS-SFs is the lack of association with real-world outcomes. The WAIS-SF for schizophrenia would be more useful if it reflected functional outcomes, such as activities of daily living or social functioning.

We have recently developed an optimal WAIS-III SF to assess current intellectual performance in patients with schizophrenia (23) according to the following five criteria: (i) prediction of FIQ, (ii) representativeness of the IQ structure, (iii) consistency of subtests across versions (WAIS-III and IV), (iv) relation to functional outcomes (daily living skills and social functioning) assessed by the UCSD Performance-based Skills Assessment [UPSA; (27)] and the Social Functioning Scale [SFS; (28, 29)], and (v) conciseness in terms of administration time. To select subtests meeting (i) and (ii) criteria, we first conducted an exploratory factor and multiple regression analyses in patients with schizophrenia, and candidate subtests were nominated to produce a candidate SF. The coverage of VIQ and PIQ and the consistency of subtests across WAIS versions, according to (iii) criterion, were also considered in the nomination process. In terms of ability to explain the variance of FIQ, correlations with functional outcomes, and time saved in comparison to full administration of the WAIS, the candidate SFs were finally examined. We found that the dyad of Similarities from verbal intellectual ability and Symbol Search from performance intellectual ability showed the highest correlations with functional outcomes and allowed the shortest administration time (Figure 1). It takes approximately 10 min to administer the WAIS-SF (Similarities and Symbol Search). It is considered that variation in processing speed is the basis of individual differences in intellectual function (13). In addition, slow cognitive processing in patients with schizophrenia is essential to the clinical manifestation of the disorder (30). Symbol Search is a constituent of the PS factor of the WAIS (Figure 1); therefore, this item is useful on the WAIS-SF for schizophrenia.

## Distribution of ID in Schizophrenia

The National Adult Reading Test (NART), the Wechsler Test of Adult Reading (WTAR), and the Wide Range Achievement Test (WRAT) scores are correlated with cognitive ability in healthy subjects, and scores on the NART, the WTAR, and the WRAT had high stability overtime (31–33). The accuracy of IQ estimates using the NART is higher than that using the WTAR (31, 32), and the WTAR is a slightly more reliable test of IQ estimates than the WRAT in a more educated and higher-functioning population (33). The NART, the WTAR, and the WRAT are three tests developed to estimate premorbid IQ because reading ability is measured as relatively intact in patients with schizophrenia (34), and its validity has been confirmed in English-speaking schizophrenia patients (13, 35, 36). The Japanese version of the NART [JART; (37)] is also widely used for Japanese-speaking patients to estimate premorbid IQ, as an equivalent to the NART (6, 7, 38–42). The stability of premorbid IQ assessed by the NART in patients with schizophrenia has been prospectively demonstrated in a longitudinal study (43). On the other hand, estimation of premorbid IQ retrospectively by these tests has a limitation. A longitudinal study design allowing long-term follow-up of high risk groups with baseline and follow-up IQ assessments is the only way to eliminate this limitation.

According to the categorization method described in previous studies (13, 44–51), patients with schizophrenia are typically classified by degree of ID into three distinct intellectual level subgroups: deteriorated, preserved, and compromised IQ.

(i) Deteriorated IQ: patients who show an ID as measured by a difference of 10 points or more between estimated premorbid and current IQ.
(ii) Preserved IQ: patients with less than a 10-point difference between estimated premorbid and current IQ and with an estimated premorbid IQ score >90.
(iii) Compromised IQ: patients with less than a 10-point difference between estimated premorbid and current IQ and with an estimated premorbid IQ below 90.

**Figure 2:**
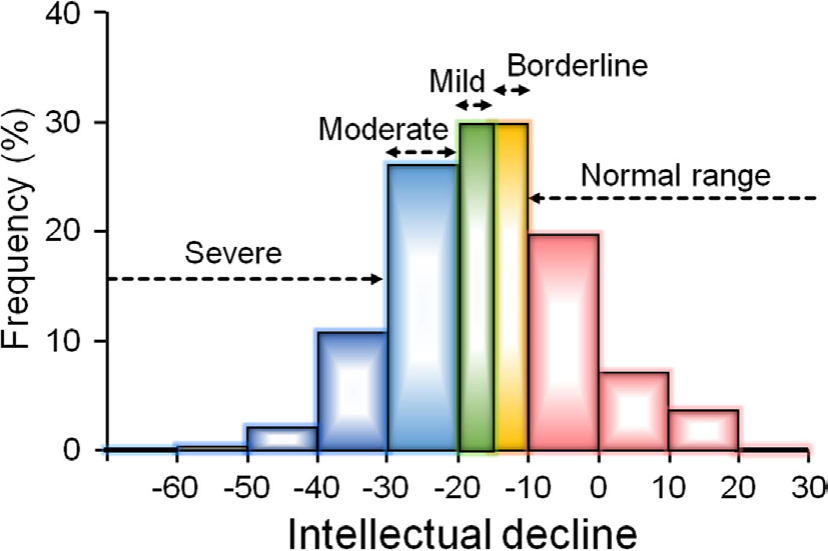
The distribution of intelligence decline (ID) in a large cohort of 446 patients with schizophrenia (18). The article by Fujino et al. (18) is published under the Attribution-Noncommercial-No Derivatives (CC BY-NC-ND) Creative Commons license, and permissions of the modification have been obtained from publisher. Approximately 30% of patients had no evidence of ID (a decline less than 10 points), while approximately 70% of patients showed deteriorated intellectual quotient: a borderline decline of 10–15 points (14%), a mild decline of 15–20 points (16%), a moderate decline of 20–30 points (26%), or a severe decline of 30 points or greater (14%).

The frequencies of preserved, deteriorated, and compromised IQ in patients with schizophrenia are summarized in Table 1 (13, 18, 44–51). For example, Weickert et al. (44) reported that 25% were categorized as having preserved IQ, 51% displayed deteriorated IQ, and 24% showed compromised IQ among 117 patients with schizophrenia. Badcock et al. (13) reported that 41% displayed preserved IQ, 43% were categorized as having deteriorated IQ, and 16% displayed compromised IQ among the 109 patients with schizophrenia. However, the distribution of ID in patients with schizophrenia was not examined in depth. Thus, we recently reported on the distribution of the ID in a large cohort of 446 patients with schizophrenia (18). Consistent with previous studies (13, 44–51), approximately 30% of patients with schizophrenia had a decline of less than 10 points, i.e., normal performance. In contrast, approximately 70% of patients showed deteriorated IQ: a severe decline of 30 points or greater (13.5%), a moderate decline of 20–30 points (26.3%), a mild decline of 15–20 points (15.9%), or a borderline decline of 10–15 points (13.7%) (Figure 2). The estimated premorbid IQ in our study (mean ± SE = 100.5 ± 0.5) was higher than those in previous studies [Weickert et al. (44), 97.1 ± 1.0: Badcock et al. (13), 95.0 ± 0.7: Leeson et al. (47), 91.0 ± 0.7]. This difference might be derived from the inclusion criteria, by which our study basically excluded schizophrenia patients who had lower estimated premorbid IQ, such as mental retardation (18), i.e., some patients with compromised IQ were initially excluded from the cohort.

**Table 1:** Frequencies of preserved, deteriorated, and compromised IQ in patients with schizophrenia.

Study name	*n*	Preserved IQ	Deteriorated IQ	Compromised IQ	Diagnostic criteria	Participants	Assessment of premorbid IQ
Weickert et al. (44)	177	24.8% (29)	51.3% (60)	23.9% (28)	DSM-III-R	SCZ	WRAT
Badcock et al. (13)	109	41.3% (45)	43.1% (47)	15.6% (17)	DSM-IV or ICD-10	SCZ	NART
Kremen et al. (46)	80	27.5% (22)	50.0% (40)	22.5% (18)	DSM-III-R	SCZ	WRAT
Potter and Nestor (49)	73	28.8% (21)	28.8% (21)	42.5% (31)	DSM-IV	SCZ or SD	WRAT
Leeson et al. (47)	129	31.0% (40)	44.2% (57)	24.8% (32)	DSM-III-R or ICD-10	first-episode SCZ or SD	WTAR
Mercado et al. (48)	149	26.8% (40)	39.6% (59)	33.6% (50)	DSM-IV	SCZ or SD	Information subtest of the WAIS-III
Ammari et al. (45)	72	44.4% (32)	36.1% (26)	19.4% (14)	DSM-IV	SCZ or SD	WRAT
Wells et al. (51)	534	29.4% (157)	44.8% (239)	25.8% (138)	DSM-IV	SCZ or SD	WTAR
Weinberg et al. (50)	96	26.0% (25)	62.5% (60)	11.5% (11)	DSM-IV	SCZ or SD	WTAR
Fujino et al. (18)	446	27.1% (121)	69.3% (309)	3.6% (16)	DSM-IV or ICD-10	SCZ	JART

*SCZ, schizophrenia; SD, schizoaffective disorder; WRAT, Wide Range Achievement Test; NART, National Adult Reading Test; WTAR, Wechsler Test of Adult Reading; WAIS, Wechsler Adult Intelligence Scale; JART, Japanese version of the NART; IQ, intellectual quotient*.

## A “Neuropsychologically Normal” Schizophrenia Group

Social/occupational dysfunction that is remarkably below the level achieved prior to the onset is listed as a diagnostic hallmark for schizophrenia in the DSM-IV and 5 criteria (52); however, neuropsychological impairments were not included in the criteria. As mentioned in the above section, no more than approximately 30% of schizophrenia patients are entirely free of neuropsychological impairments, although there is heterogeneity in this proportion among studies (13, 18, 44, 46, 47, 53, 54). If cognitive impairments are a core feature of schizophrenia, it may be difficult to explain the presence of neuropsychologically normal function in schizophrenia patients who display the full clinical syndrome.

A cluster-analysis approach can group patients on the basis of profiles or patterns of cognitive impairments and produce more homogeneous groupings (1, 55), providing an opportunity to classify patients. Cluster-analysis studies of cognitive function within schizophrenia patients have successfully created meaningful subgroups with at least three clusters: patients who are neuropsychologically normal, patients with intermediate cognitive deficits, and patients with widespread deficits (1, 54–60). One consideration is that schizophrenia patients who have mild ID or are neuropsychologically normal may be a unique subtype and may comprise a relatively benign subtype of schizophrenia in terms of prognosis. This type of patient may be better educated and/or have higher premorbid IQ than patients with impaired cognitive function. Indeed, some studies have indicated that patients with preserved IQ tended to be better educated and/or show higher premorbid IQ than patients with deteriorated or compromised IQ (13, 44, 47), while other studies have not indicated such associations (46).

**Figure 3:**
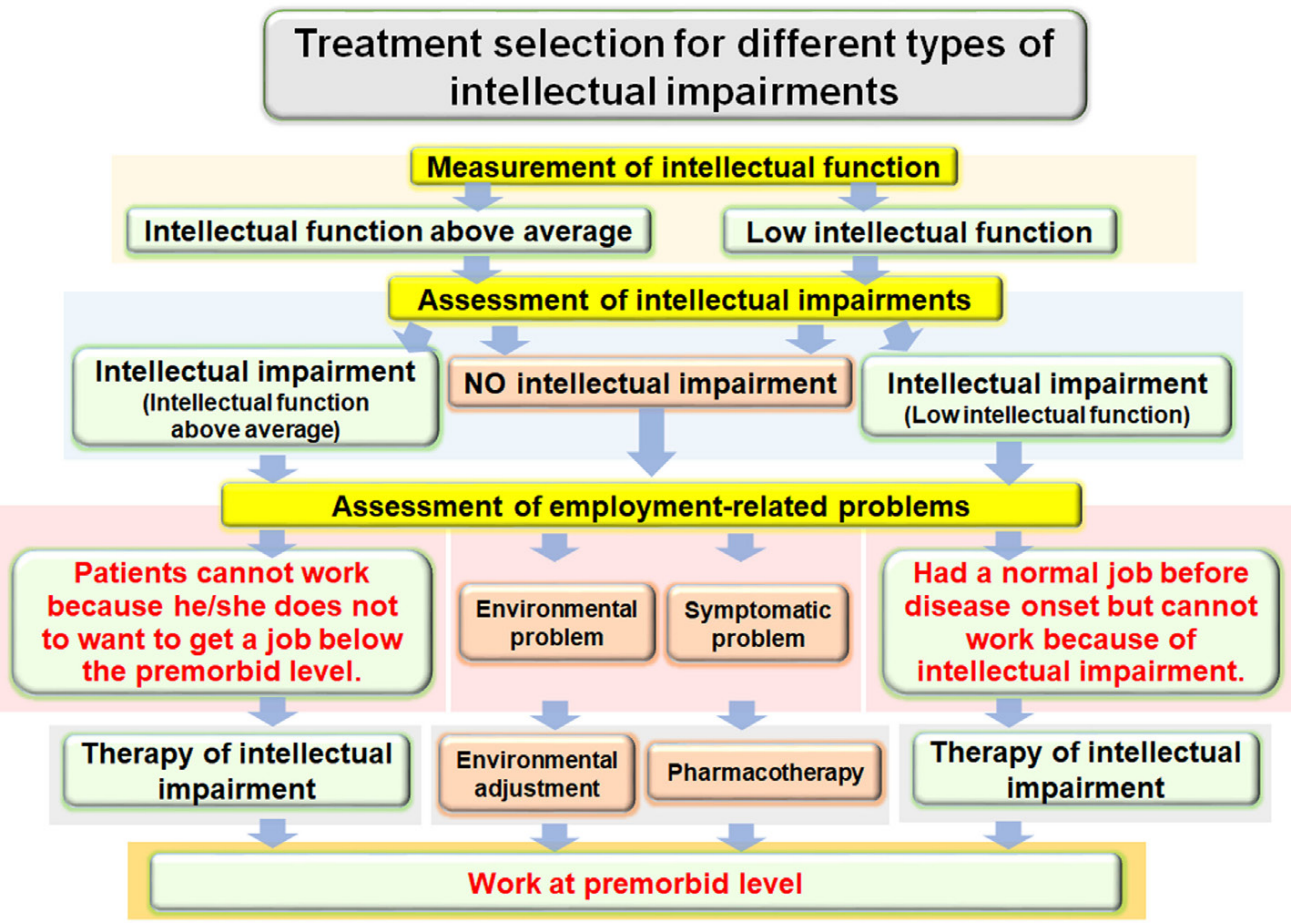
Algorithm for treatment of intellectual impairments in patients with schizophrenia to improve employment outcomes.

On the other hand, schizophrenia patients with preserved IQ exhibited specific deficits in at least some cognitive domains, especially executive function and attention, compared with healthy subjects who had similar IQ, even though that subset of patients had apparently normal current intellectual function (13, 44, 53, 61, 62). These studies suggest that ID, although typical of schizophrenia, is not universally characteristic and that executive function and attention deficits may be core features of schizophrenia, independent of intelligence variations (44). However, not only IQ decline but also executive function and attention deficits may be less than universally characteristic of patients with schizophrenia because the disorder is clinically heterogeneous. We suggest that it is necessary to make a detailed, personalized assessment of cognitive impairments, such as IQ decline and executive function and attention deficits, in order to treat functional impairments appropriately in schizophrenia patients. As shown in Figure 3, we additionally suggest an algorithm to be employed in the treatment of intellectual impairments in schizophrenia patients. First, we measure intellectual function in patients with schizophrenia using estimated premorbid and current IQ, and we assess whether the patient has intellectual impairment. According to the status of intellectual impairment, we assess employment-related problems in each patient and treat those problems. The ultimate goal is for the patient to work at a premorbid level.

## Pathophysiology of Cognitive Impairments in Schizophrenia

Schizophrenia has a strong genetic basis with an estimated heritability of approximately 80% (63). Most cognitive functions also have a genetic component and are heritable (*h*^2^ = 0.33–0.85) (64–68). Impairments such as cognitive decline are stable, partly affected by antipsychotic medications (47, 69–71), and typically stronger in schizophrenia patients (72). Cognitive dysfunctions have also been shown in the unaffected relatives or twin siblings of people with schizophrenia (73). Previous genome-wide association studies (GWASs) on schizophrenia and cognitive functions have indicated that many genes or genetic variants mediate both cognitive function and the risk of schizophrenia (7, 17, 73–77). These previous GWASs on schizophrenia and cognitive function have explained up to approximately 20% of the genetic architecture of risk for schizophrenia and poor cognitive function (74–77). In addition, a part of the phenotypic correlation between cognitive function and schizophrenia results from identical genetic effects (73, 77). Polygenic risk scores for cognitive dysfunction were associated with a higher risk of schizophrenia, whereas polygenic risk scores for schizophrenia were associated with lower cognitive ability (73, 77, 78). Thus, cognitive functions have been proposed as a useful intermediate phenotype (39, 79–82) to understand the genetic mechanisms involved in the pathophysiology of schizophrenia.

We suggest that genetic variants related to cognitive impairments including ID might be associated with the *N*-methyl-d-aspartate (NMDA) glutamate network (7) or in delta(4)-desaturase, sphingolipid 2 (*DEGS2*) gene expression (17, 83). Glutamate is the major excitatory neurotransmitter of the central nervous system (CNS) and is involved in basic neuronal functions and CNS processes, including memory, learning, and synaptic plasticity (84). Decreased function of glutamate transmission through NMDA receptors that are voltage-dependent ionotropic glutamate receptors has been involved in the pathophysiology of schizophrenia (85). NMDA receptor antagonists, including phencyclidine and ketamine, can induce schizophrenia-like psychotic symptoms and cognitive impairments in individuals without schizophrenia and exacerbate symptoms in schizophrenia patients (86, 87). Schizophrenia patients have aberrant density and subunit composition of NMDA receptors in the postmortem brains (88, 89).

From birth onward, the *DEGS2* gene is most abundantly expressed in the dorsolateral prefrontal cortex (DLPFC) that is a major component of the high-order associative cortex related to both schizophrenia and cognitive functions (83, 90). Carriers of the ID-associated risk allele had lower *DEGS2* expression than subjects homozygous for the non-risk allele in the DLPFC (17, 83). The *DEGS2* enzyme is implicated in the biosynthesis of phytosphingolipids. Sphingomyelin is a type of sphingolipid, and abnormalities of the sphingomyelin can cause several CNS diseases, including schizophrenia (91, 92). The low expression pattern of *DEGS2* is correlated with the low distribution of phytosphingolipids (93, 94). The *DEGS2* risk polymorphism related to low *DEGS2* expression in the DLPFC may be associated with lower synthesis of sphingolipids in the brain because *DEGS2* mRNA expression regulates synthesis of phytosphingolipids during keratinocyte differentiation (93). Further research is needed to clarify the role of glutamate network and *DEGS2* gene expression in the pathogenesis of ID of schizophrenia.

## Effects of Antipsychotics on ID in Schizophrenia

Positive symptoms and negative symptoms have become targets for medication in patients with schizophrenia. However, based on the evidence that the functional disability accompanying schizophrenia is strongly associated with cognitive impairments and is not correlated with psychotic symptoms (12), we should focus on ID in patients with schizophrenia. The therapeutic effects of antipsychotics are predominantly limited to the positive and negative symptoms, and those drugs have substantially less impact on improvement of cognitive impairments. It has been reported that typical antipsychotics are applied without regard for cognitive impairments in patients with schizophrenia and do little to improve them (84, 95), whereas atypical antipsychotics have been reported to partly reduce cognitive impairment in schizophrenia patients (84, 95, 96). Atypical antipsychotics are superior to typical antipsychotics at improving cognitive impairment (effect size = 0.24), although there are no differences in improvement among atypical antipsychotics (97). Such improvements are also observed in specific studies of first-episode schizophrenia and early-onset schizophrenia (98–100).

To date, the mechanisms whereby antipsychotics act on ID have remained unclear. Atypical antipsychotics produce extensive blockade of serotonin (5-HT)_2A_ receptors, direct or indirect stimulation of 5-HT_1A_ receptors, and, to a lesser extent, a reduction in dopamine D2 receptor-mediated neurotransmission (101–103). The serotonergic actions of the atypical antipsychotics are able to mitigate cognitive impairments in patients with schizophrenia (103). In addition, 5-HT_6_ or 5-HT_7_ receptor antagonists may also contribute to the beneficial effects of the antipsychotics on cognitive function (103).

Furthermore, anticholinergic load is related to lower cognitive function in schizophrenia patients (104, 105). The administration rate of anticholinergic medications is lower in patients who are prescribed atypical antipsychotics compared with those who are prescribed typical antipsychotics, supporting the idea that cognitive improvements would differ between users of typical and atypical antipsychotics. The discontinuation of long-term anticholinergic use would mitigate cognitive impairment in patients with schizophrenia (105, 106). In addition, the use of benzodiazepines is related to cognitive impairments in schizophrenia patients (107, 108). The reduction or discontinuation of long-term benzodiazepines with atypical antipsychotics ameliorates cognitive impairments in patients with schizophrenia (108). These findings suggest that the use of anticholinergics and long-term benzodiazepines would be related to cognitive impairments in patients with schizophrenia. Therefore, we suggest that physicians should prescribe only atypical antipsychotics, without anticholinergics or benzodiazepines, to reduce the cognitive impairments observed in schizophrenia. On the other hand, the use of benzodiazepines and anticholinergics would treat unwanted symptoms, such as anxiety and extrapyramidal symptoms, in schizophrenia. The development of novel antipsychotics that are unlikely to result in extrapyramidal symptom or treat anxiety symptom is warranted.

The cognitive impairments observed in schizophrenia may be affected by decreased activity of the M1 muscarinic acetylcholine receptor, dysfunction of NMDA glutamatergic neurotransmission, and serotonergic dysregulation. However, the effects of cholinesterase inhibitors, antidepressants, or 5-HT2 antagonists as adjunctive treatments to antipsychotics for cognitive impairments in schizophrenia have been limited (109–113). Approval of antipsychotic drugs with novel mechanisms of action has been rare in recent years despite extensive efforts by investigators. Further investigations are essential to address this issue by identifying new pharmacological targets related to ID in patients with schizophrenia. We suggest that patients without ID should be initially detected and excluded from putative clinical trials of drugs meant to mitigate ID in patients with schizophrenia.

## Cognitive Remediation

Cognitive remediation or cognitive rehabilitation interventions are designed to improve cognitive impairments through repeated practice of cognitive tasks and/or strategy training. As the effects of antipsychotics on cognitive impairments in schizophrenia patients have been limited, a number of cognitive remediation programs have been increasingly examined to improve cognitive impairments (114). Randomized controlled studies have shown cognitive remediation to have positive effects on cognitive impairments in patients with first-episode psychosis as well as schizophrenia (114–118). The average effect size was small to moderate at approximately 0.40 (114, 115). Some types of cognitive remediation involve extensive use of computers, while others focus primarily on paper-and-pencil tasks. The Neuropsychological Educational Approach to Remediation (NEAR) is an evidence-based cognitive remediation approach (114). The NEAR program involves a combination of “drill and practice” exercises and teaching strategies to ameliorate cognitive impairments (114). NEAR utilizes commercially available educational software to create a rich learning environment that is intrinsically motivating and rewarding (114). Cognitive remediation interventions are conducted individually or in groups. Although the goal of cognitive remediation is to ameliorate cognitive impairments in patients with schizophrenia, more than 12.0% of participants dropped out at different points during the program (114, 115). Therefore, it may be difficult to generalize a cognitive remediation as a treatment for cognitive impairments in patients with schizophrenia. In addition, it remains unclear whether the improvements are sustained or temporary, although short-term effects of cognitive remediation on cognitive function have been indicated (115). Similar to our proposal regarding the composition of drug trials, we suggest that patients without ID should be initially detected and excluded from clinical trials to develop cognitive remediation programs for patients with schizophrenia.

## Conclusion

In this study, we reviewed the literature of ID in patients with schizophrenia. Although intellectual impairments are a core feature of schizophrenia, the effects of antipsychotics and cognitive remediation against those impairments have been limited. We have held several workshops on the brief assessment of ID in schizophrenia to promote the concept of monitoring ID in Japanese patients with schizophrenia. Further studies are warranted to develop novel antipsychotics and cognitive remediation for patients with ID.

## Author Contributions

RH supervised the entire project and was critically involved in the design, analysis, and interpretation of the data. KO, CS, and TS collected the data, wrote the manuscript, and were responsible for performing the literature review. HF, YY, HY, and MF were heavily involved in the collection of the majority of the data and contributed intellectually to the interpretation of the data. All authors contributed to and have approved the final manuscript.

## Conflict of Interest Statement

The authors declare that the research was conducted in the absence of any commercial or financial relationships that could be construed as a potential conflict of interest.
